# Epigenetic small-molecule screen for inhibition and reversal of acinar ductal metaplasia in mouse pancreatic organoids

**DOI:** 10.3389/fphar.2024.1335246

**Published:** 2024-03-06

**Authors:** Kalina R. Atanasova, Corey M. Perkins, Ranjala Ratnayake, Jinmai Jiang, Qi-Yin Chen, Thomas D. Schmittgen, Hendrik Luesch

**Affiliations:** ^1^ Department of Medicinal Chemistry, College of Pharmacy, University of Florida, Gainesville, FL, United States; ^2^ Center for Natural Products, Drug Discovery and Development, College of Pharmacy, University of Florida, Gainesville, FL, United States; ^3^ Department of Pharmaceutics, College of Pharmacy, University of Florida, Gainesville, FL, United States

**Keywords:** pancreatic cancer, acinar ductal metaplasia, epigenetics, drug screening, organoids, marine natural product, largazole and largazole homodimer

## Abstract

**Background:** Acinar ductal metaplasia (ADM) is among the earliest initiating events in pancreatic ductal adenocarcinoma (PDAC) development.

**Methods:** We developed a novel morphology-based screen using organoids from wildtype and *p48^Cre/+^* (Cre) mice to discover epigenetic modulators that inhibit or reverse pancreatic ADM more effectively than the broad-spectrum HDAC inhibitor trichostatin A (TSA).

**Results:** Of the 144 compounds screened, nine hits and two additional natural product HDAC inhibitors were validated by dose-response analysis. The class I HDAC inhibitors apicidin and FK228, and the histone methyltransferase inhibitor chaetocin demonstrated pronounced ADM inhibition and reversal without inducing significant cytotoxicity at 1 µM. Thioester prodrug class I HDAC inhibitor largazole attenuated ADM while its disulfide homodimer was effective in both ADM inhibition and reversal. Prioritized compounds were validated for ADM reversal in *p48^Cre/+^*; LSL-*Kras^G12D/+^* (KC) mouse organoids using both morphological and molecular endpoints. Molecular index analysis of ADM reversal in KC mouse organoids demonstrated improved activity compared to TSA. Improved prodrug stability translated into a stronger phenotypic and molecular response. RNA-sequencing indicated that angiotensinogen was the top inhibited pathway during ADM reversal.

**Conclusion:** Our findings demonstrate a unique epigenetic mechanism and suggest that the phenotypic screen developed here may be applied to discover potential treatments for PDAC.

## 1 Introduction

Pancreatic ductal adenocarcinoma (PDAC) is among the most lethal cancers worldwide (Siegel et al., 2023) with the majority of cases being discovered after metastasis has occurred (Adamska et al., 2017; Sakamoto et al., 2020; Kim, 2022). The dire outcomes are in great part a lack of effective treatments and poor methods of early detection. Understanding the early stages of PDAC development is critical to improve early detection and treatment strategies. Studies in genetically modified mouse models reveal an underlying role for pancreatic acinar cells in PDAC development (Grippo et al., 2003; Desai et al., 2007; Guerra et al., 2007; Habbe et al., 2008). One of the earliest known initiating events for PDAC is the process of acinar ductal metaplasia (ADM) (Liou et al., 2015; Johnson et al., 2019). During ADM, acini transdifferentiate into duct-like structures with reduced expression of acinar markers such as amylase (AMY2A) or carboxypeptidase (CPA2) and increased ductal markers such as cytokeratin 19 (KRT19). Following inflammatory or other insults, ADM is a natural process that occurs to protect acinar cells from further enzymatic damage. In mouse models of PDAC, ADM is achieved by restricted expression of *Kras* in acinar cells (Habbe et al., 2008; Kopp et al., 2012), experimental pancreatitis (Strobel et al., 2007), TGF-α (Bockman and Merlino, 1992) or other factors that activate EGFR (Liou et al., 2015). ADM is believed to be irreversible with respect to mutant *Kras* (Morris et al., 2010; Krah et al., 2015; Chuvin et al., 2017).

The current FDA-approved therapies for PDAC include gemcitabine in combination with platinum agents or the FOLFIRINOX drug combination (National Cancer Institute, 2023). Many drugs in clinical trials for treating PDAC such as romidepsin (FK228), vorinostat (SAHA) or curcumin (Nebbioso et al., 2012; Xu et al., 2022) rely on epigenetic mechanisms to induce cell death of tumor cells. However, despite initial successful effects in clinical trials, many of these epigenetic drug trials have been terminated due to high toxicity (Nebbioso et al., 2012).

Using primary cultures of mouse pancreas at different days of embryonic development, treatment with the histone deacetylase (HDAC) inhibitor trichostatin A (TSA) reduced acinar differentiation and promoted ductal differentiation (Haumaitre et al., 2008). Treatment of cultured acinar cells with TSA reduced caerulein-induced trypsin activation (Hartman et al., 2015). Using the caerulein-induced pancreatic injury model in mice, treatment with the HDAC inhibitor valproic acid delayed recovery of the pancreas, reduced acinar cell proliferation, maintaining ADM and thus delaying acinar redifferentiation (Eisses et al., 2015). HDAC expression, in particular class I HDAC, was upregulated during caerulein-induced pancreatitis in mice and inhibition of class I HDAC with MS-275 reduced ADM both *in vitro* and *in vivo* (Bombardo et al., 2017). We recently showed that TSA or the STAT3 inhibitor LLL12B inhibited ADM formation in a mouse acinar organoid 3D culture model (da Silva et al., 2022). Moreover, TSA exposure following completion of ADM induced phenotypic and gene expression changes reminiscent of ADM reversal in organoids from mice carrying the *Kras* ^*G12D*^ mutation (da Silva et al., 2022). *KRAS* ^*G12D*^ is among the most common mutations observed in PDAC patients and is considered to be one of the main drivers of ADM and progression to PDAC (Ying et al., 2016; Guo et al., 2020; Gu et al., 2021; Rozengurt and Eibl, 2021; He et al., 2022).

Given the need for new, effective therapeutics with minimal toxicity, we developed a novel medium-throughput screen to discover epigenetic modulating compounds that inhibit and/or reverse ADM. We utilized the mouse acinar organoid 3D-culture model and monitored ADM or its reversal using high-content organoid morphology as a readout. We screened the Cayman’s small-molecule epigenetic modulator library (ESL) which contains 144 compounds with the goal to identify important regulators of ADM with therapeutic potential. A large component of the ESL library (43 compounds) consists of HDAC inhibitors with broad or narrow molecular target and isoform range. Of these, 34 HDAC inhibitors were Zn^2+^ -dependent (targeting HDACs class I, II or IV) with differential class or isoform selectivity (class I vs. II vs. I/II) selectivity and nine were NAD^+^-dependent (targeting class III HDACs).

During the initial screen, we identified nine epigenetic modulating compounds, two of which were class I HDAC inhibitors, that induced phenotypic changes with high mechanism-dependent selectivity and low cytotoxicity. The effects of these nine compounds and additional class I-selective HDAC inhibitor prodrugs, largazole (thioester), largazole homodimer (disulfide), and the functionally similar natural product FK228 were further validated in dose-response studies using the same model. The prioritized compounds were effective at re-expression of acinar genes (*Amy2a, Cpa2*) and suppressing ductal genes (*Krt19*, *Sox9*) in the *Kras* ^*G12D*^ mouse organoid model demonstrating that ADM is reversible even in the context of mutant *Kras*.

## 2 Materials and methods

### 2.1 Mice

C57BL/6J (wild type) mice were used in ADM inhibition assays. *p48^Cre/+^* mice (Cre-mice) were bred to LSL-*Kras^G12D/+^* to produce *p48^Cre/+^*; LSL-*Kras^G12D/+^* mice (KC mice). Genotyping for the presence of the transgene was performed by Transnetyx (Cordova, TN).

### 2.2 Mouse acinar ductal metaplasia culture

Acinar cells were isolated as previously reported (da Silva et al., 2022) from the pancreas of 6–8-week-old wild type (ADM inhibition assays), Cre-mice (ADM reversal assays), or KC mice (ADM reversal assays). Following a 30-min digestion with collagenase P, enzyme inactivation with fetal bovine serum containing Hanks’ balanced salt solution, and multiple centrifugation steps, cells were passed through a series of strainers (500, 300, and 200 µm). The resulting pellet was resuspended in media to the desired density to yield 50 organoids/well with growth factor reduced Matrigel (Corning). The mixture was maintained on ice during the pipetting steps. 20 µL of the mixture was pipetted into a 384-well CellCarrier Ultra plates (PerkinElmer), followed by 30 min of incubation to solidify the mixture and then 40 µL of warm media was added. The organoids seeded in Matrigel were left overnight to acclimate and then subjected to treatment or left to further develop ADM before treatment.

### 2.3 Compound screen, reagents, treatment and sample processing

#### 2.3.1 ADM inhibition and reversal experimental design

In ADM inhibition experiments wild type mouse organoids were imaged live after overnight acclimation using the Operetta High Throughput Screen imaging system (PerkinElmer) using ×20 objective, brightfield filter and 15 fields of view/well covering ∼80% of the well area. The wells were immediately treated using a JANUS liquid handling system (PerkinElmer) and a 200 nL pin tool. In ADM reversal experiments *p48^Cre/+^* (screen and validation experiments) or KC-mice organoids (follow up experiments) were left to incubate at 37°C, 5% CO_2_ for an additional 72 or 48 h, respectively, until ducts formed, then fresh media was exchanged and the organoids were imaged and treated in same manner as for ADM inhibition experiments.

#### 2.3.2 Epigenetic compound treatment

For screening experiments, organoids were treated with the Cayman small-molecule epigenetic library (Cayman Chemicals, #11076) at final concentration of 1 µM. For validation studies, organoids were treated with a relevant concentration range of 3-fold dilutions of the selected compounds. Vehicle control wells (0.5% DMSO, *n* = 4) were included on every plate. TSA (Sigma-Aldrich, #T8552) was used as a positive control based on previous findings (da Silva et al., 2022) and was tested in a 3-fold dose range from 32 nM to 10 µM (quadruplicate wells per dose). FK228 (romidepsin) was purchased from Sigma-Aldrich, (#SML1175). Largazole (Chen et al., 2018) and largazole homodimer (Salvador et al., 2014) were synthesized as previously described.

#### 2.3.3 Viability staining and imaging

Post treatment plates were incubated at 37°C, 5% CO_2_ for 72 h and the wells were visualized for duct/cluster-like morphology. Media was removed and organoids in Matrigel were washed once with 100 µL/well of Dulbecco’s phosphate buffered saline (DPBS, Corning), followed by staining with Calcein AM (Sigma) in DPBS at final concentration of 4 µM for 1.5 h at 37°C in the dark. After staining, organoids were washed again with 100 µL/well of DPBS and left in fresh DPBS for live imaging. Organoids were imaged on the Operetta High-Throughput Screen imaging system using ×20 objective, brightfield and green fluorescence filters at the same 15 fields/well that were imaged before the treatment.

#### 2.3.4 Duct/cluster-like counting

Following 72 h of treatment, total and live (Calcein AM positive) duct and cluster counts were compared to the vehicle control treated wells to determine cytotoxicity. Brightfield (both before treatment and 72 h post treatment) and green-fluorescence filter (at 72 h post treatment) images were analyzed by identification of the primary objects in stitched images of the 15 fields/well using a pixel classification of background/objects-based procedure in Ilastik 1.3.2 (Berg et al., 2019) and a custom-built pipeline in CellProfiler 3.1.8 software (McQuin et al., 2018), followed by object quantification, and viability quantification (using the green channel, calcein AM staining images). Objects were classified as either duct-like or cluster-like using the machine learning object classification software CellProfiler Analyst 3.0.3 (Jones et al., 2009) based on the properties/features collected from the CellProfiler software. All stitched images were also visually assessed to confirm observations from the machine learning components and the robustness of the pipeline/classification process, as well as to detect morphological changes that did not fall within the duct-like/cluster-like classification, e.g., duct size changes and other morphological changes. ADM at 72 h post treatment was expressed as the percent ratio of viable ducts and viable acinar clusters from all live objects for both the ADM inhibition and the ADM reversal screens. Analysis of experiments carried out for the selected few hits done in KC mice was performed manually by visualization due to the developed pipeline not being able to recognize texture and morphological differences specific for the cyst-like organoids compared to the acinar clusters.

### 2.4 RNA isolation and quantitative gene expression

To process the 384 well plates for qRT-PCR for consistency, we used a modification of our method to remove Matrigel from the cell mixture (Da Silva et al., 2020). Media was removed by inverting the plate onto paper towels to blot off the media. The plate was then placed on ice for 10 min to liquefy the Matrigel. Forty µL of ice-cold PBS was added to each well of the plate on ice. The contents of 16 wells were then combined per treatment by removing 60 µL from each well and combined into a 2 mL centrifuge tube on ice. An additional 1 mL of cold PBS was added to the tube and resuspended. The tubes were centrifuged at 1,000 × G for 5 min at 4°C. The supernatant was removed by pipetting and then resuspended in 2 mL of cold PBS, followed by an additional centrifugation step at 1,200 × G for 5 min at 4°C. Supernatant was removed by pipetting and the cell pellet was resuspended in 700 µL of TRIzol reagent. Total RNA was isolated using the miRNeasy protocol (Qiagen). Sixty ng of total RNA was converted to cDNA in a 20 µL RT reaction using random primers and MMLV reverse transcriptase (Thermo). qPCR was performed using the QuantStudio™ 7 Flex Real-Time PCR System (Thermo). Data are presented using the 2^−ΔΔCT^ method (Livak and Schmittgen, 2001) relative to vehicle control and normalized to 18S rRNA. Primer sequences have been previously published (da Silva et al., 2022).

### 2.5 RNA sequencing and pathway enrichment analysis

Illumina RNA library construction was performed at the Interdisciplinary Center for Biotechnology Research (ICBR) Gene Expression Core, University of Florida (UF). RNA quantitation was done on a NanoDrop Spectrophotometer (NanoDrop Technologies, Inc.), and sample quality was assessed using the Agilent 2100 Bioanalyzer (Agilent Technologies, Inc.). SMART-Seq V4 ultra low input RNA kit were used for RNAseq library construction according to the user manual. Illumina sequencing libraries were generated with 125 pg of cDNA using Illumina Nextera DNA Sample Preparation Kit (Cat#: FC-131-1024) according to manufacturer’s instructions. The libraries were pooled in equal molar concentration. Normalized libraries were submitted to the “Free Adapter Blocking Reagent” protocol (FAB, Cat# 20024145) in order to minimize the presence of adaptor-dimers and index hopping rates. The library pool was diluted to 0.8 nM and sequenced on one S4 flow cell lane (2 × 150 cycles) of the Illumina NovaSeq6000 using NovaSeq Control Software v1.6. Sample sequencing was performed at the ICBR NextGen Sequencing (https://biotech.ufl.edu/next-gen-dna/, RRID:SCR_019152). Additional details on library generation and sequencing are given in the Supplementary Material.

Reads acquired from the Illumina NovaSeq 6000 platform were cleaned up with the cutadapt program (Martin, 2011) to trim the sequencing adaptors and low-quality bases with a quality phred-like score <20. Reads <40 bases were excluded from RNA-seq analysis. The genome of *Mus musculus* (version GRC38, mm10) from the Ensembl database was used as the reference sequences for RNA-seq analysis and cleaned reads were mapped to the reference sequences using the read mapper of the STAR package (Spliced Transcripts Alignment to a Reference, v2.7.9a) (Dobin et al., 2013). The mapping results were processed with the HTSeq (High-Throughput Sequence Analysis in Python, v0.11.2) (Anders et al., 2015), samtools, and scripts developed in house at ICBR to remove potential PCR duplicates and count uniquely mapped reads for gene expression analysis. Outliers were detected using PCA analysis and volcano plot analysis based on all identified genes using R-package (v4.1.3). The gene express levels were analyzed by a DESeq2-based R pipeline.

Gene expression levels of approximately 4,000 differentially expressed genes compared to vehicle control at 72 h post treatment were compared using Ingenuity Pathway Analysis software (QIAGEN Inc., https://digitalinsights.qiagen.com/IPA) and hits were considered as significant when they passed the following cut-offs: fold change difference greater than 1.5 and a false discovery rate (FDR) *p*-value of less than 0.05. The z-score was calculated dependent upon the fold change and FDR requirements, resulting in the identification of the top upstream and downstream effectors following reversal treatment.

### 2.6 Statistical analysis and selection of compounds for validation

Percent live ducts for each treatment was compared to vehicle control and *p* values were calculated using two-tailed Student’s t-test with unequal variances. The Z′-score for evaluating the optimization quality of the assay was calculated using negative control (vehicle only: DMSO, 0.5%) and positive control at concentration inducing inhibition or reversal of ADM in over 80% of all organoids in the well (TSA, 10 µM) (Zhang et al., 1999). Significance was considered at *p* ≤ 0.05. Compounds that showed significant decrease in % live ducts compared to vehicle control, while showing over 50% total viable objects, were selected for further validation and analysis. The ADM Reversal Index (ADMRI) is defined as the mean fold change in expression (treated *versus* control, 72 h post treatment) as determined by qRT-PCR of acinar genes (*Amy2a*, *Cela*, and *Cpa2*) divided by the mean fold change in ductal gene (*Krt19*, *Krt7*, and *Sox9*) expression (Eq. 1). We annotated two sets of genes previously associated with acinar/ductal phenotype of pancreas or genes associated with onset or progression of PDAC, from the literature (Benitz et al., 2019; Qadir et al., 2020). Volcano plots were constructed from the changes in expression of 27 acinar and 23 ductal/PDAC genes following ADM reversal as previously described (Jiang et al., 2023). A list of the acinar and ductal/PDAC genes may be found in Supplementary Table S1 (Benitz et al., 2019; Qadir et al., 2020).

### 2.7 Ingenuity pathway analysis

Gene expression levels of approximately 4,000 differentially expressed genes compared to vehicle control at 72 h post treatment for ADM reversal in KC mouse organoids were compared using Ingenuity Pathway Analysis software (QIAGEN Inc., https://digitalinsights.qiagen.com/IPA). Top hits were ranked by the following criteria. Only the following molecular types were included, growth factor, cytokine, group, transcription regulator and complex. The top upstream regulators were ranked based on the z-score. A positive z-score indicates activation while a negative z-score indicates inhibition.

## 3 Equations

### 3.1 ADM reversal index (ADMRI)

$$ADM\;Reversal\;Index\;\left(ADMRI\right)=\frac{\text{Mean Fold Change of Acinar Genes}\;\left(Amy2a,Cela1,Cpa2\right)}{\text{Mean Fold change of Ductal Genes}\;\left(Krt19,Krt7,Sox9\right)}$$ (1)

## 4 Results

### 4.1 Assay validation

**FIGURE 1:**
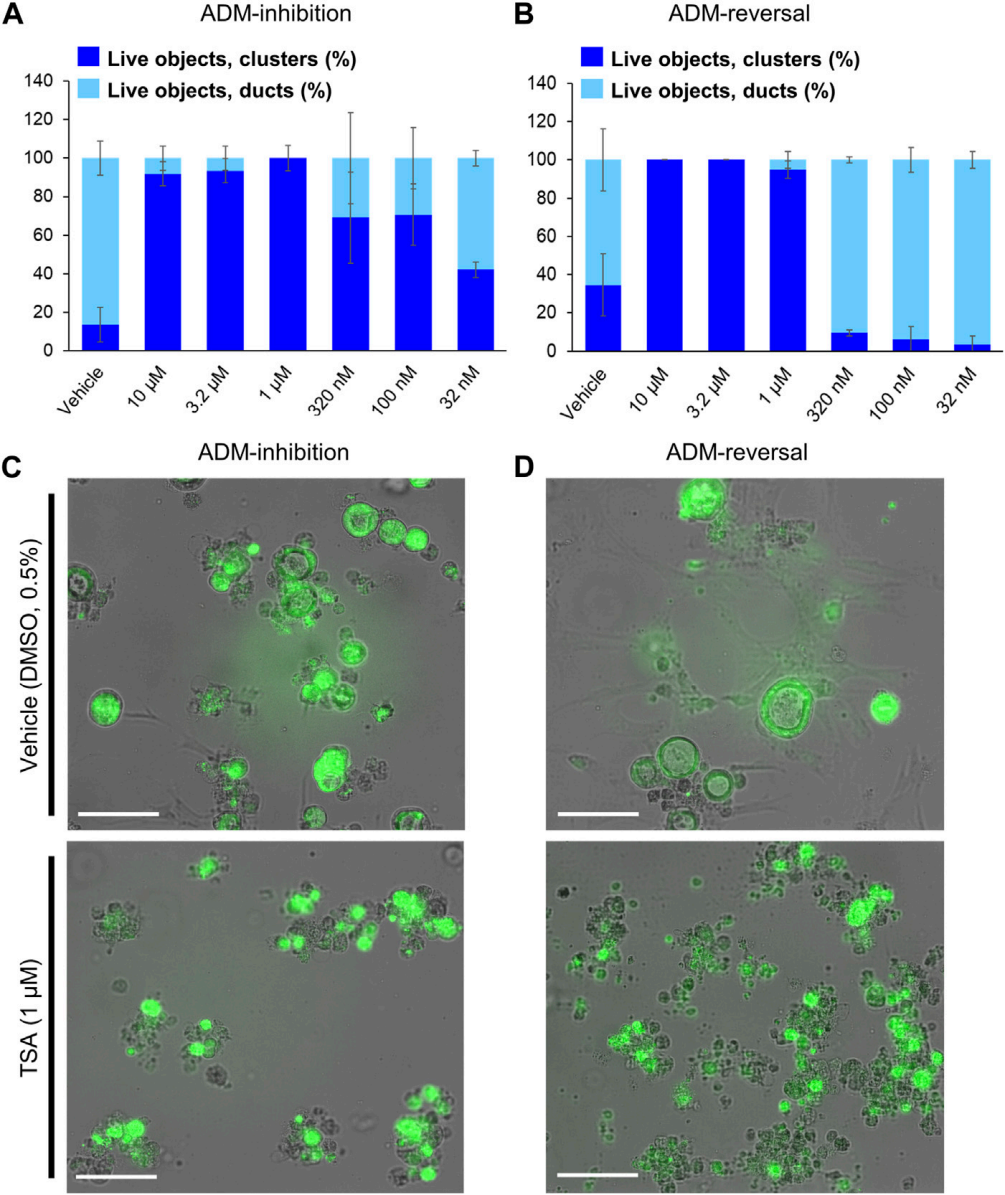
Distribution of viable ducts and acinar clusters in trichostatin A (TSA)-treated organoids. Percent duct/cluster distribution (± SD) of live objects at 72 h post treatment with positive control TSA as a function of the concentration in ADM inhibition (wildtype mouse organoids, Z’ = 0.61) **(A)** and ADM reversal (Cre-mice organoids, Z’ = 0.56) **(B)** assays. Representative images at 1 µM TSA for ADM inhibition **(C)** and ADM reversal **(D)** assays. Scale bars represent 100 µm. Single biological replicate of quadruplicate technical replicates, mean ± SD.

Experimental efficiency was verified in both the inhibition (wildtype mouse organoids) and reversal (*p48* ^*Cre*^ (Cre) mouse organoids) screening modes using TSA as the positive control. A concentration-dependent relationship was observed for both inhibition and reversal effects while cell morphology showed the presence of viable acinar clusters rather than viable ducts as with the vehicle (0.5% DMSO) controls (Figure 1). The Z′ for both the ADM inhibition (Z′ = 0.61) and ADM reversal (Z′ = 0.56) was calculated using the positive control TSA. The values for Z′ was >0.50 indicating that both assays were sufficiently optimized for high-throughput screening.

### 4.2 Epigenetic small-molecule library screen (ADM inhibition)

**FIGURE 2:**
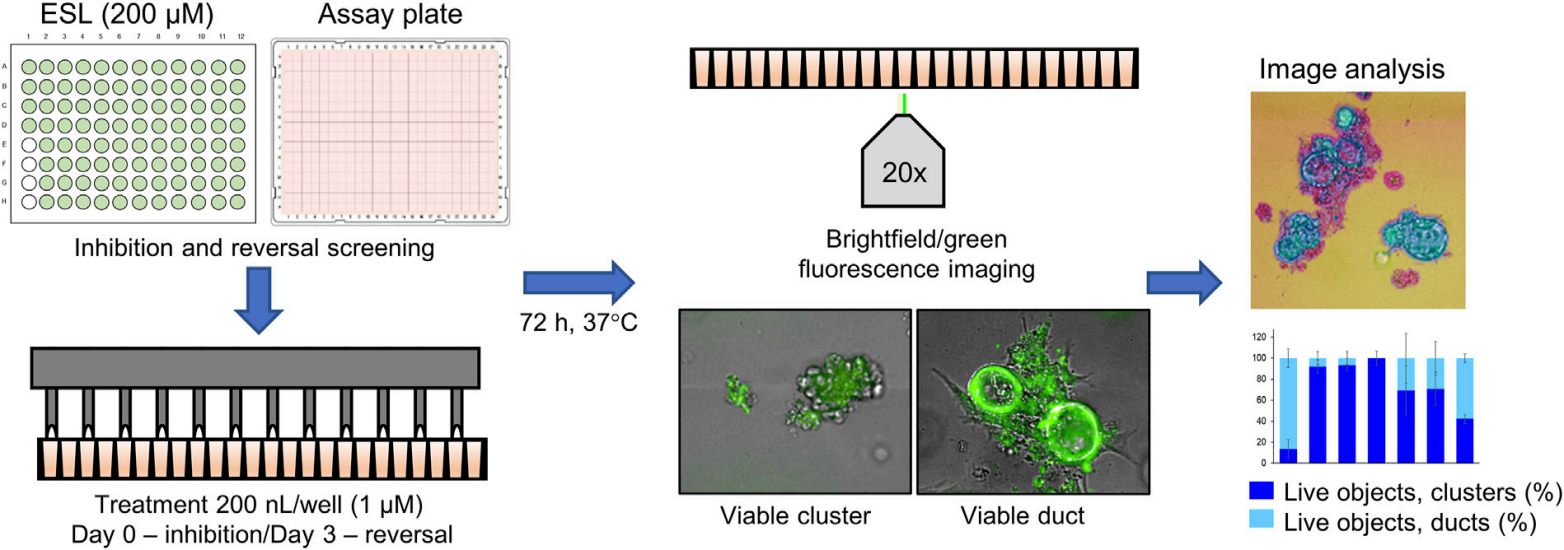
Schematic representation of the ADM screen. Seeded organoids originating from wildtype (WT) or *p48^Cre/+^* (Cre) mice were treated with the Cayman small-molecule epigenetic modulator library (ESL) at final concentration of 1 µM using an automated dispensing system and left to incubate for 72 h. Following incubation, organoids were stained using the calcein AM viability dye and imaged using a high throughput imaging system at ×20 magnification to obtain high resolution images for further image analysis.

**FIGURE 3:**
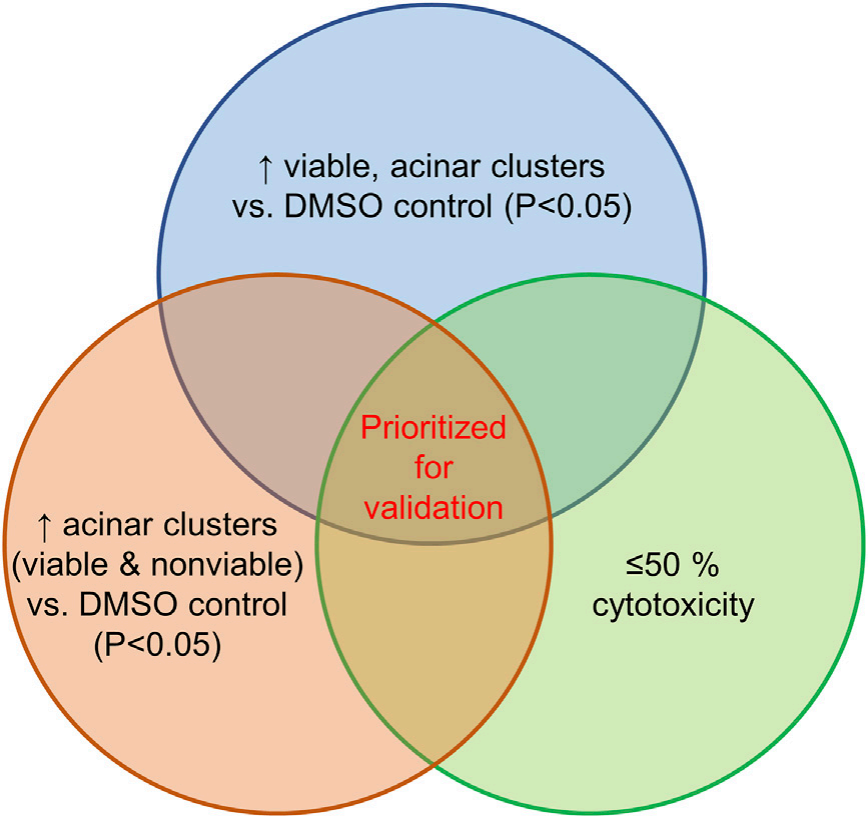
Selection criteria for compound validation. Summary of selection criteria applied to prioritize compounds for validation from ADM inhibition (wildtype mice organoids) and ADM reversal (Cre-mice organoids) screens. Cytotoxicity was evaluated by calcein AM staining. Compounds that overlapped in all three selection criteria (apicidin and chaetocin in ADM inhibition assay; chaetocin in ADM reversal assay) are shown in red and were prioritized for validation in dose response.

The screen of 144 epigenetic modulating compounds for ADM inhibition was performed on wildtype mouse organoids. Schematic representation of the screening workflow is shown in Figure 2. Results from the inhibition screening assays were subjected to three criteria in order to select hits for further validation (Figure 3): *i*) compounds that showed a higher percentage of viable acinar clusters compared to the vehicle control (*p* < 0.05), *ii*) compounds that showed a higher percentage of total clusters (viable and nonviable) compared to the vehicle control (*p* < 0.05), and *iii*) the compounds that satisfied the criteria *i*) and *ii*) and produced ≤ 50% cytotoxicity 72 h post treatment as measured by calcein AM staining (for detailed results see Supplementary Figures S1–S4).

**FIGURE 4:**
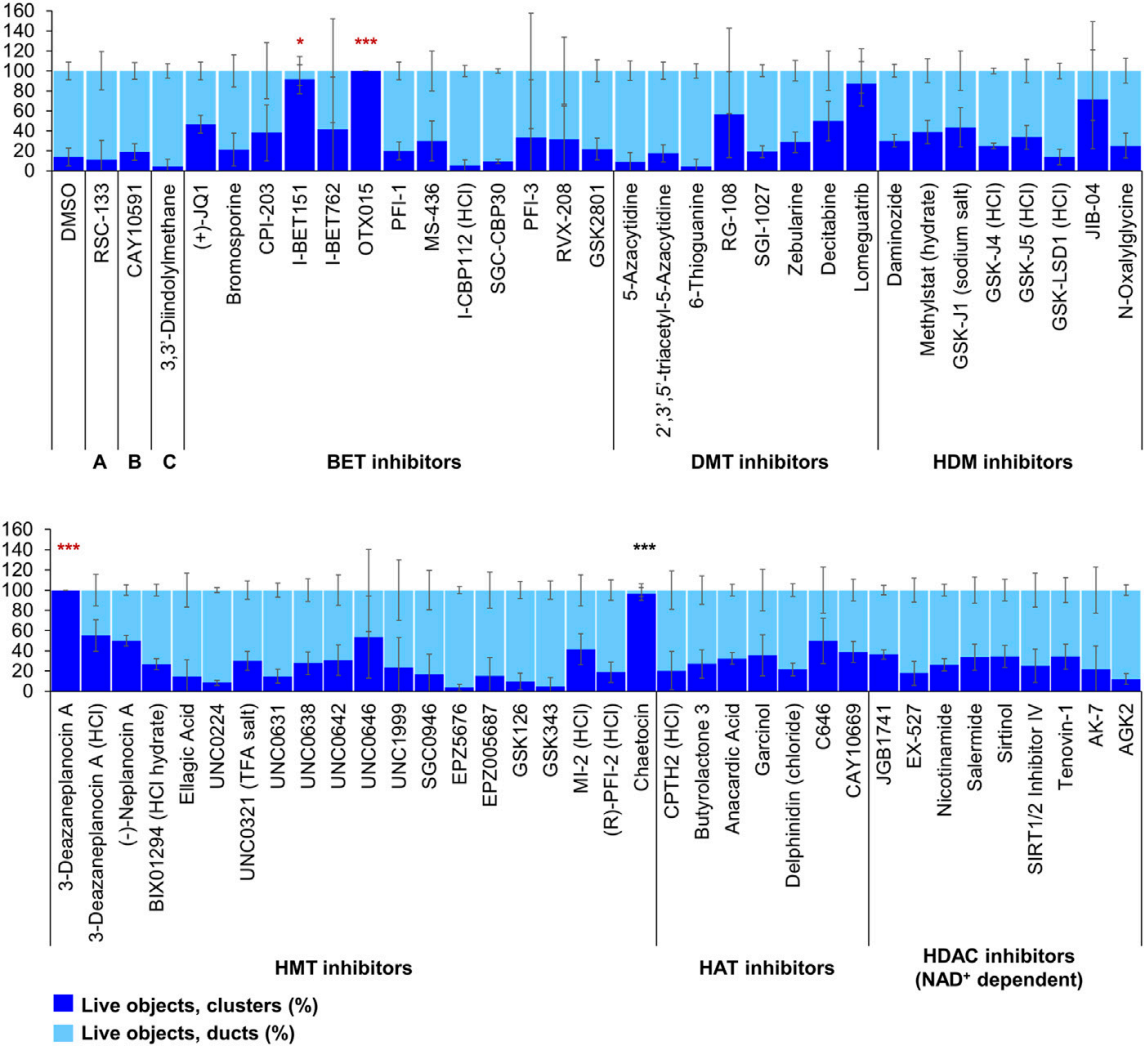
ADM inhibition from epigenetic compound library screen (compounds 1–68 grouped by target). The ADM inhibition assay screen was performed in duplicate using the Cayman ESL on wildtype mouse organoids. The mean percentage of viable ducts and acinar clusters (± SD) 72 h post treatment. BET = bromodomain and extraterminal domain inhibitors; DMT = DNA methyltransferase inhibitors; HDM = histone demethylase inhibitors; HMT = histone methyltransferase inhibitors; HAT = histone acetyltransferase inhibitors; HDAC = histone deacetylase inhibitors (NAD^+^ dependent); HCl = hydrochloride; TFA salt = trifluoroacetic acid salt. *p* values were calculated using two-tailed Student’s *t*-test with unequal variances. Significance was accepted at *p* ≤ 0.05 only when averages of clusters are higher than the respective vehicle control. *p-value = 0.05–0.01; **p-value = 0.01–0.001; ***p-value < 0.001. Red asterisks denote compounds that showed significantly higher cluster/duct ratios of live objects but had overall less than 50% viability of total objects (false positive). Black asterisks denote compounds that showed significantly higher cluster/duct ratios of live objects and more than 50% of total objects were viable by calcein AM staining (true positive). Single biological replicate of quadruplicate technical replicates, mean ± SD.

**FIGURE 5:**
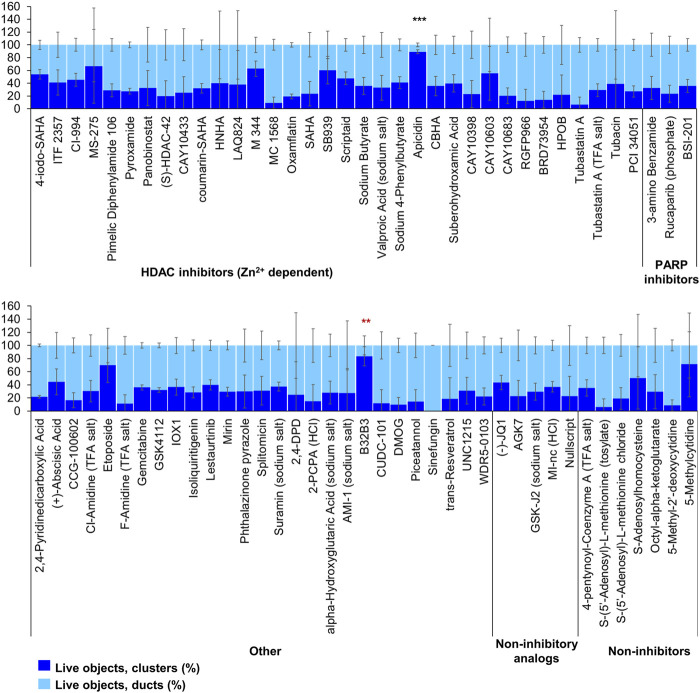
ADM inhibition from epigenetic compound library screen (compounds 69–144 grouped by target). The ADM inhibition assay screen was performed in duplicate using the Cayman ESL on wildtype mouse organoids. The mean percentage of viable ducts and acinar clusters (± SD) 72 h post treatment. HDAC = histone deacetylase inhibitors (Zn^2+^ dependent); PARP = poly-ADP ribose polymerase inhibitors; HCl = hydrochloride; TFA salt = trifluoroacetic acid salt. *p* values were calculated using two-tailed Student’s t-test with unequal variances. Significance was accepted at *p* ≤ 0.05 only when averages of clusters are higher than the respective vehicle control. *p-value = 0.05–0.01; **p-value = 0.01–0.001; ***p-value < 0.001. Red asterisks denote compounds that showed significantly higher cluster/duct ratios of live objects but had overall less than 50% viability of total objects (false positive). Black asterisks denote compounds that showed significantly higher cluster/duct ratios of live objects and more than 50% of total objects were viable by calcein AM staining (true positive). Single biological replicate of quadruplicate technical replicates, mean ± SD.

Of the 144 compounds screened for ADM inhibition, six compounds showed significantly higher percentages of live clusters at the tested concentration of 1 µM compared to the vehicle control (chaetocin, apicidin, IBET151, OTX015, 3-deazaneplanocin A and B32B3). Four of those compounds (IBET762, OTX015, 3-deazaneplanocin A and B32B3) resulted in >50% cytotoxicity based on the calcein AM staining (Figures 4, 5; Supplementary Figures S1, S3). The two prioritized compounds from the inhibition screen are apicidin (Zn^2+^ dependent, class I HDAC inhibitor) and the histone methyltransferase (HMT) inhibitor chaetocin (Figures 4, 5), fulfilling our criteria.

### 4.3 Epigenetic small-molecule library screen (ADM reversal)

**FIGURE 6:**
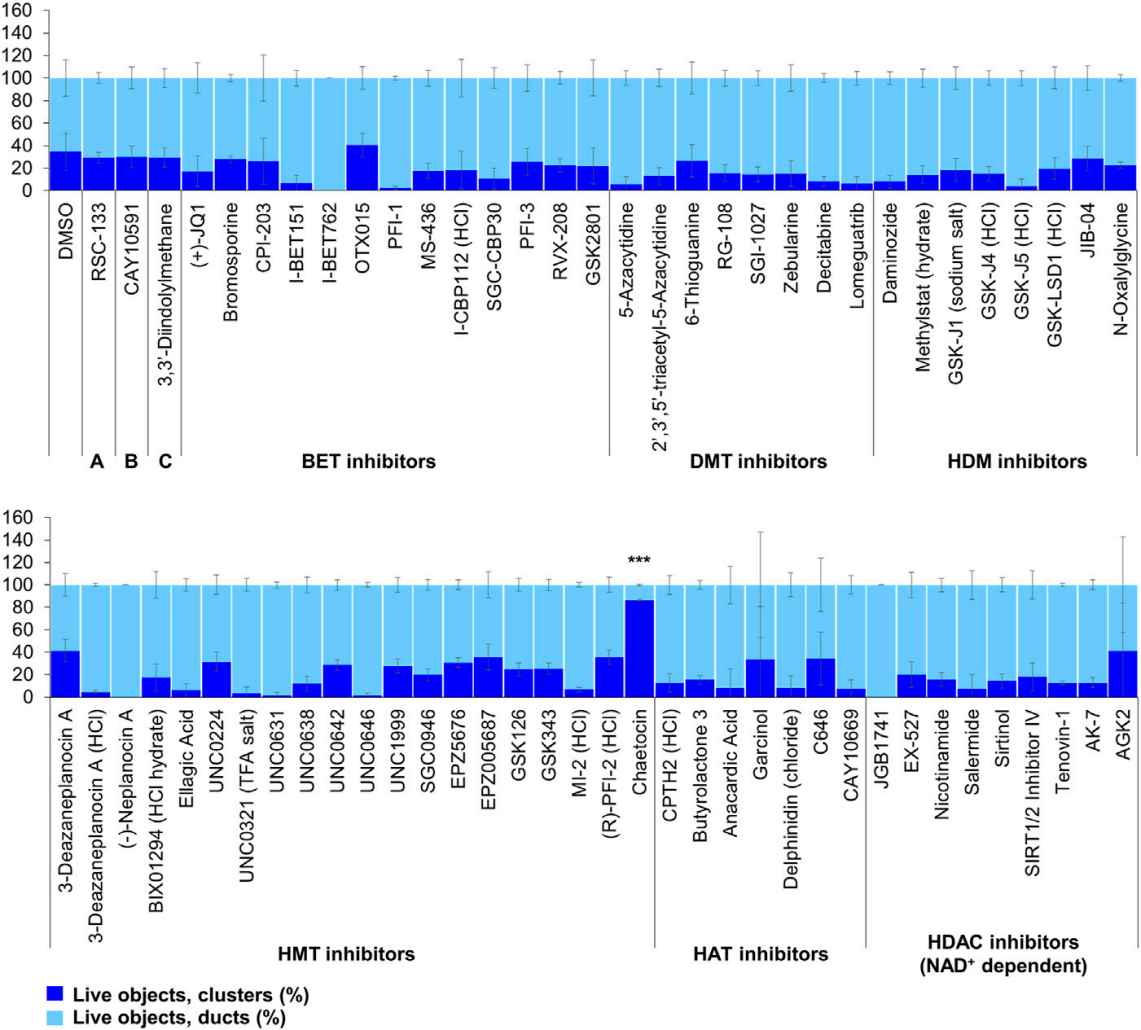
ADM reversal from epigenetic compound library screen (compounds 1–68 grouped by target). The ADM reversal assay screen was performed in duplicate using the Cayman ESL on Cre mouse organoids. The mean percentage of viable ducts and acinar clusters (± SD) 72 h post treatment. BET = bromodomain and extraterminal domain inhibitors; DMT = DNA methyltransferase inhibitors; HDM = histone demethylase inhibitors; HMT = histone methyltransferase inhibitors; HAT = histone acetyltransferase inhibitors; HDAC = histone deacetylase inhibitors (NAD^+^ dependent); HCl = hydrochloride; TFA salt = trifluoroacetic acid salt. *p* values were calculated using two-tailed Student’s t-test with unequal variances. Significance was accepted at *p* ≤ 0.05 only when averages of clusters are higher than the respective vehicle control. *p-value = 0.05–0.01; **p-value = 0.01–0.001; ***p-value < 0.001. Black asterisks denote compounds that showed significantly higher cluster/duct ratios of live objects and more than 50% of total objects were viable by calcein AM staining (true positive). Single biological replicate of quadruplicate technical replicates, mean ± SD.

**FIGURE 7:**
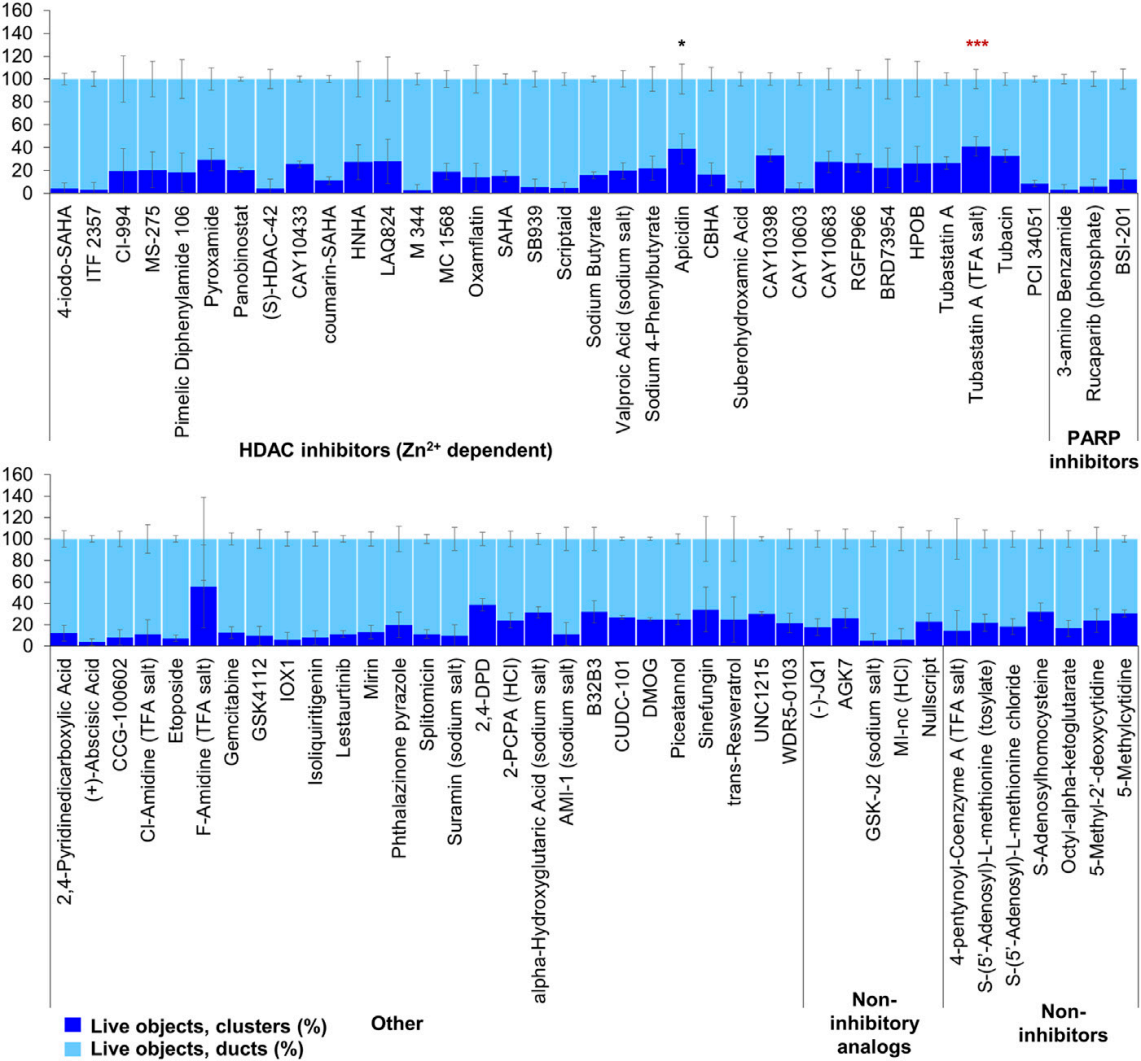
ADM reversal from epigenetic compound library screen (compounds 69–144 grouped by target). The ADM reversal assay screen was performed in duplicate using the Cayman ESL on Cre mouse organoids. The mean percentage of viable ducts and acinar clusters (± SD) 72 h post treatment. HDAC = histone deacetylase inhibitors (Zn^2+^ dependent); PARP = poly-ADP ribose polymerase inhibitors; HCl = hydrochloride; TFA salt = trifluoroacetic acid salt. *p* values were calculated using two-tailed Student’s t-test with unequal variances. Significance was accepted at *p* ≤ 0.05 only when averages of clusters are higher than the respective vehicle control. *p-value = 0.05–0.01; **p-value = 0.01–0.001; ***p-value < 0.001. Red asterisks denote compounds that showed significantly higher cluster/duct ratios of live objects but had overall less than 50% viability of total objects (false positive). Black asterisks denote compounds that showed significantly higher cluster/duct ratios of live objects and more than 50% of total objects were viable by calcein AM staining (true positive). Single biological replicate of quadruplicate technical replicates, mean ± SD.

The screen of 144 epigenetic modulating compounds for ADM reversal was performed on Cre mouse organoids. The criteria to prioritize compounds from the screen were identical to those described for ADM inhibition. Only two compounds (chaetocin and apicidin) passed our rigorous selection criteria (Figures 6, 7). The class II HDAC (HDAC6) inhibitor tubastatin A passed criteria *i* and *ii* however it induced >50% cytotoxicity (Figures 6, 7, Supplementary Figures S2, S4).

**FIGURE 8:**
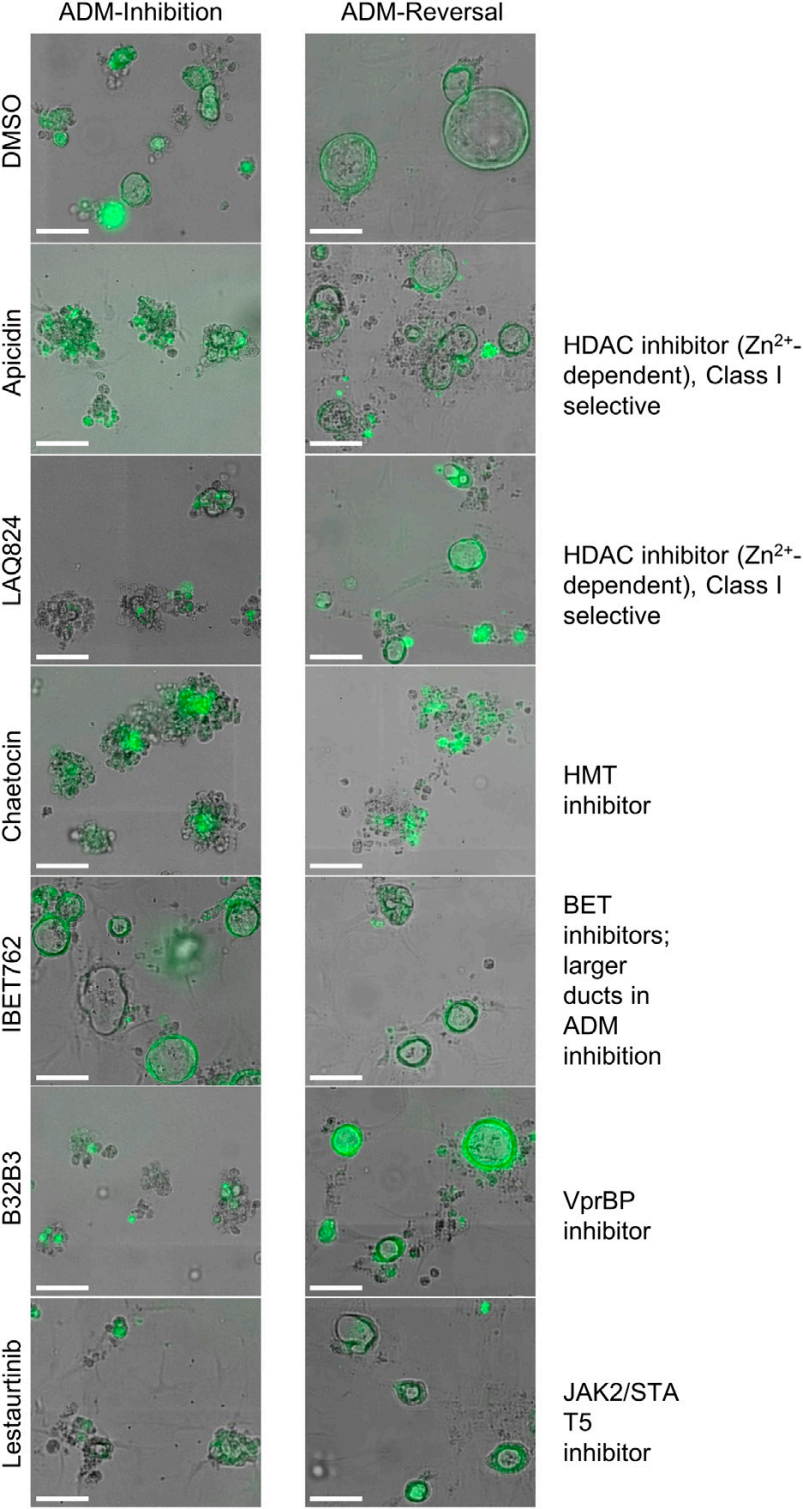
Cellular morphology of organoids following epigenetic small-molecule modulator library treatment in ADM inhibition (wildtype mice) or reversal (Cre mice) assays. Representative images of observed range of organoid morphology changes after treatment with epigenetic small-molecule modulator library compounds at 1 µM for 72 h in ADM inhibition (left) or ADM reversal (right). Size markers represent 100 µm.

In addition to the automated, quantitative data obtained from the screen, we visually observed the sets of images collected from each of the treatments for both inhibition and reversal to see if any compounds produced unique morphological characteristics that could not be detected by our pipeline (Table 1; Figure 8). LAQ824 reduced duct size during inhibition (Figure 8). The BET inhibitors IBET762 and IBET151, produced notably enlarged ducts during ADM inhibition but not during ADM reversal (Figure 8).

**TABLE 1:** Summary of observed behavior and compound targets of Cayman Epigenetic Screening Library showing distinct morphological effects on ducts in the ADM inhibition (clusters to ducts) and/or ADM reversal (reversing ducts to clusters) assays in comparison to vehicle control (0.5% DMSO).

Compound (1μM)	ADM inhibition	ADM reversal	Major drug target
Apicidin	Did not form ducts	Reduced duct sizes	HDACs (Zn^2+^ dependent), class I selective
LAQ824	Small ducts	No change	HDACs (Zn^2+^ dependent), class I selective
Chaetocin	Did not form ducts	Complete reversal to clusters	HMTs
Lestaurtinib	Small ducts	Reduced duct sizes	JAK2, STAT5, STAT3
B32B3	Did not form ducts	No change	VprBP
IBET762	Enlarged ducts	Reduced duct sizes	BETs
IBET151	Enlarged ducts	Reduced duct sizes	BETs
(+) JQ1	Enlarged ducts	Reduced duct sizes	BETs
PFI-1	Enlarged ducts	No change	BETs

### 4.4 Concentration-effect analysis for selected compounds

Based on our observations from the initial screens, a small number of compounds were selected for further validation (summarized in Table 1). The four bromodomain inhibitors IBET151, IBET762, (+)-JQ1 and PFI-1, that showed pronounced enlargement of formed ducts compared to those observed in the vehicle control were selected for further verification by concentration-effect analysis (Figure 8). The compounds LAQ824 (dacinostat, class I/II HDAC inhibitor) and lestaurtinib (tyrosine kinase inhibitor), showed only pronounced reduction in duct size (Figure 8), but not reversal to acinar morphology, and was therefore selected for follow up to see if reversal would be achieved at slightly higher concentrations. Also selected for concentration-effect analysis were apicidin and chaetocin as they were the prioritized compounds in both the inhibition and reversal screens.

**FIGURE 9:**
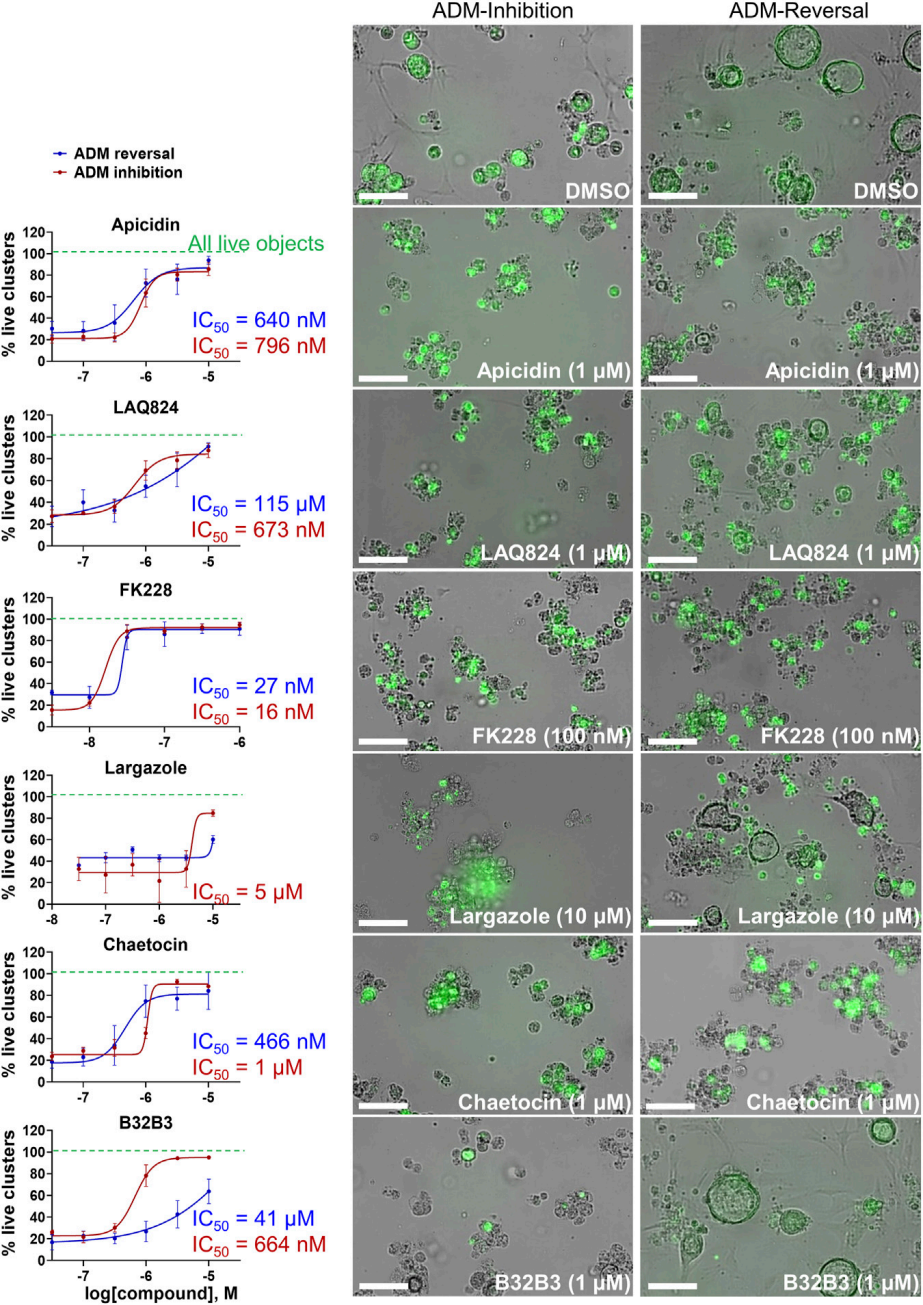
Validation of selected hits in dose response at 72 h post treatment. (Left) Percent live clusters from all live objects in organoids treated with dose response of select hits in ADM inhibition (wildtype mice) and ADM reversal (Cre mice) assay modes. IC_50_ values were calculated using GraphPad Prism software. (Right) Representative images of morphological changes observed with hit compounds at the lowest dose which caused morphologically distinguishable effects in ADM inhibition and ADM reversal assay modes. Size markers represent 100 µm. Single biological replicate of quadruplicate technical replicates, mean ± SD.

The concentration-effect analysis was performed in wildtype (inhibition) and Cre (reversal) mouse organoids using concentrations ranging from 32 nM to 10 µM. Since apicidin and the positive control (trichostatin A) are both Zn^2+^-dependent HDAC inhibitors with differential selectivity profile (apicidin: class I; TSA: class I/II) and to possibly elucidate mode of action, we included in the follow up studies two class I-selective HDAC inhibitors: FK228, an FDA-approved anticancer drug derived from terrestrial bacteria, and largazole, a preclinical stage marine natural product (Hong and Luesch, 2012) (Figure 9). Both of these compounds are class I HDAC inhibitors predominantly targeting HDACs 1, 2, and 3 and were not part of the ESL (Hong and Luesch, 2012). In terms of ADM reversal, FK228 was the most efficacious with low nM IC_50_, followed by apicidin, chaetocin and LAQ824; B32B3 was ineffective at reversing ADM (Figure 9). Of the six compounds selected for ADM inhibition, FK228 was the most potent with an IC_50_ of 16 nM (Figure 9). This was followed by apicidin, chaetocin, LAQ824 and B32B3 which were equipotent at IC_50_ ranging from ∼0.5 to 1 µM (Figure 9). Chaetocin was the only HMTase inhibitor compound from the library that showed similar effects in both the ADM inhibition (IC_50_ ∼ 1 µM) and the ADM reversal (IC_50_ ∼ 0.5 µM) assays. Compounds that did not validate with the initially observed results in either or both assay modes are shown in Supplementary Figure S5. From the BET inhibitors selected for validation due to the unique effects, IBET762 (at 10 μM–32 nM) (Figure 8) and IBET151 (at 10 μM–100 nM) and (+) JQ1 (at 10 μM–1 μM) (data not shown) showed enlargement of ducts in the ADM-inhibition assay, but no clear reversal in the ADM-reversal assay, and therefore we did not continue further analysis with these compounds. PFI-1 (the fourth BET inhibitor) did not show any effects in either mode during validation (data not shown).

**FIGURE 10:**
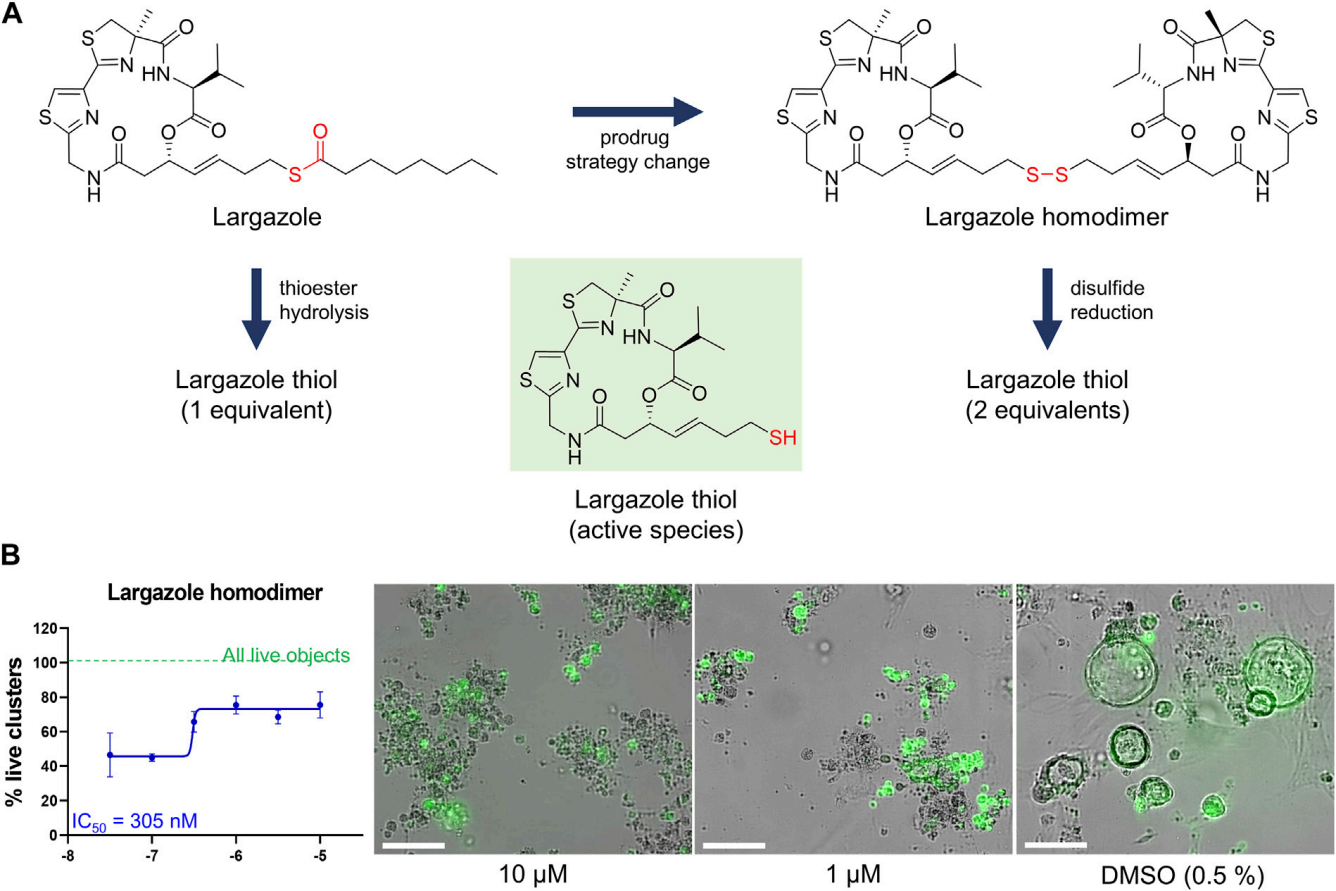
Testing of potency of largazole homodimer in ADM reversal mode (Cre mice organoids) at 72 h post treatment. **(A)** Structures of Largazole homodimer and justification for drug selection. Prodrug strategy was applied, which increases stability of the compound and also generates two equivalents of the active species, leading to increased potency compared to the parent compound. **(B)** Dose response study of largazole homodimer in ADM reversal assay mode. (Left) Percent live clusters from all live objects. IC_50_ value was calculated using GraphPad Prism 9 software based on quadruplicate experiments. (Right) Representative images of morphological changes observed with the homodimer at select doses in ADM reversal assay mode compared to vehicle control (0.5% DMSO). Scale bars represent 100 µm. Single biological replicate of quadruplicate technical replicates, mean ± SD.

Although largazole has been previously shown to have similar potency to FK228 in enzyme and cell based (proliferation) assays in colorectal cancer HCT116 cells (Hong and Luesch, 2012), it was less efficacious at both inhibiting and reversing ADM compared to FK228 (Figure 9). This may be due to its lower stability in the extracellular matrix where it can be hydrolyzed before entry into the cells and therefore would not reach the target (Liu et al., 2010; Hong and Luesch, 2012). Largazole is a thioester prodrug that undergoes protein-assisted hydrolysis to liberate the active species, largazole thiol (Hong and Luesch, 2012), while the disulfide FK228 is reductively activated in the cell, mediated by glutathione (Liu et al., 2010). We have previously shown that the timing of prodrug activation for largazole can be modulated using disulfide homodimer and heterodimers (Salvador et al., 2014). Therefore, we performed an additional test in the ADM reversal mode assay with a largazole homodimer that was designed to have improved stability while also liberating two equivalents of active species, largazole thiol (Liu et al., 2010) (Figure 10). The homodimer showed similar efficacy during ADM reversal compared to apicidin, with an IC_50_ near 1 μM, but still 10-fold less activity compared to FK228 (IC_50_ near 0.1 µM) (Figures 9, 10).

### 4.5 ADM reversal in KC mouse organoids

**FIGURE 11:**
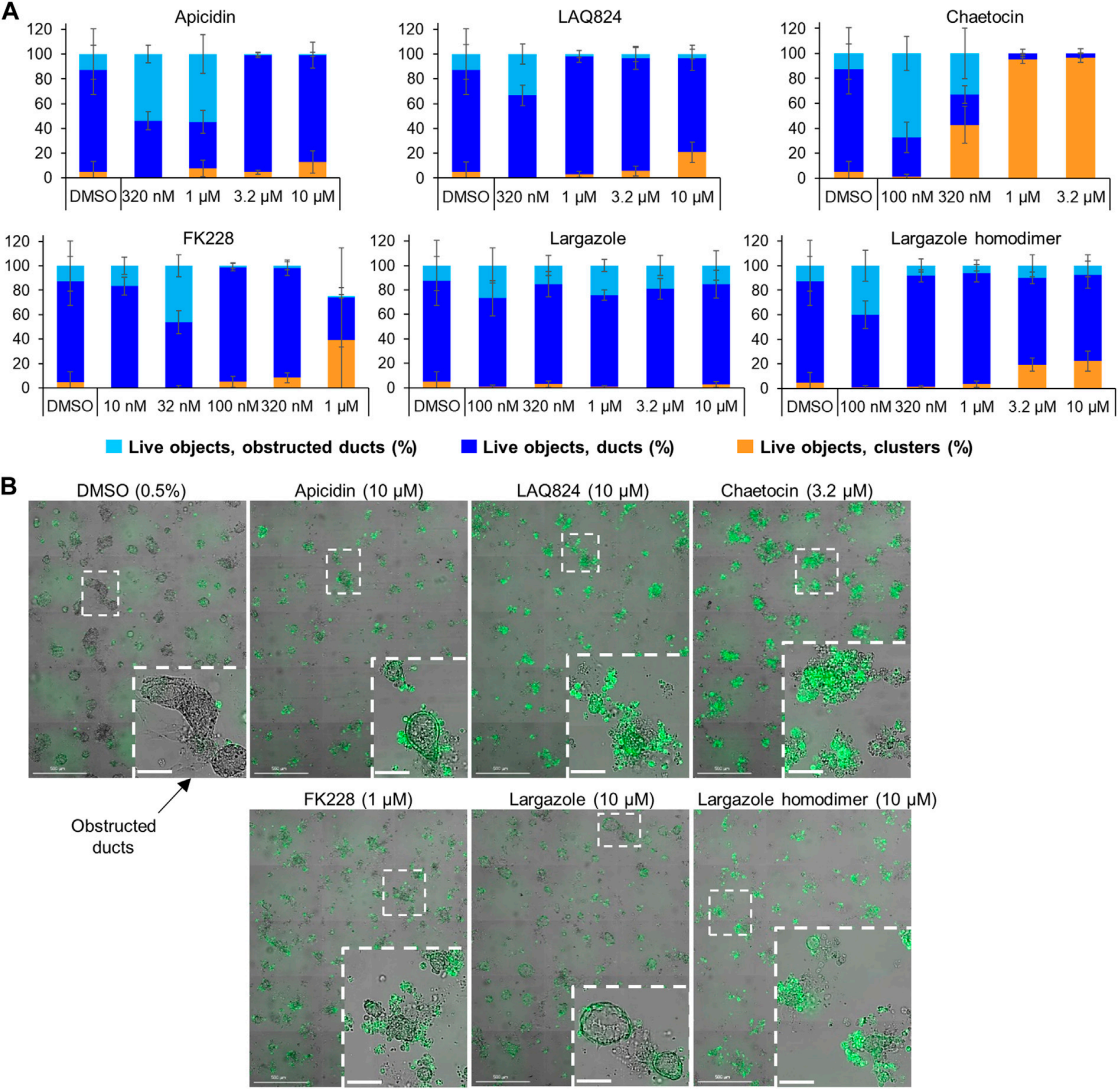
Effects of select validated compounds in KC mouse acini, ADM reversal assay. **(A)** Percent live clusters from all live objects in organoids treated with a small dose range of the compounds. **(B)** Representative images and enlarged area of the observed morphological effects. Size markers for full well images represent 500 µm and enlarged areas 100 µm.

The validated compounds from both assays at 1 µM or lower were also tested for ADM reversal in the more clinically relevant *Kras* ^G12D^-mutant mouse model (KC mice). These mice carry the *KRAS* ^*G12D*^ gene mutation that is present in PDAC patients. Cultured KC organoids develop ADM faster than wild-type or Cre mouse organoids (Mizutani et al., 2010; Fan et al., 2018). In the KC mice, chaetocin concentrations of 1 and 3.2 µM were the most effective at reversing acinar morphology (Figure 11). In the vehicle control-treated organoids, the ducts progressed into obstructed ducts (cyst-like) that lacked a visible lumen (Giri et al., 2017). Higher concentrations of the compounds apicidin, LAQ824, largazole homodimer and FK228 prevented the formation of these cyst-like structures compared to the DMSO control (Figure 11).

**FIGURE 12:**
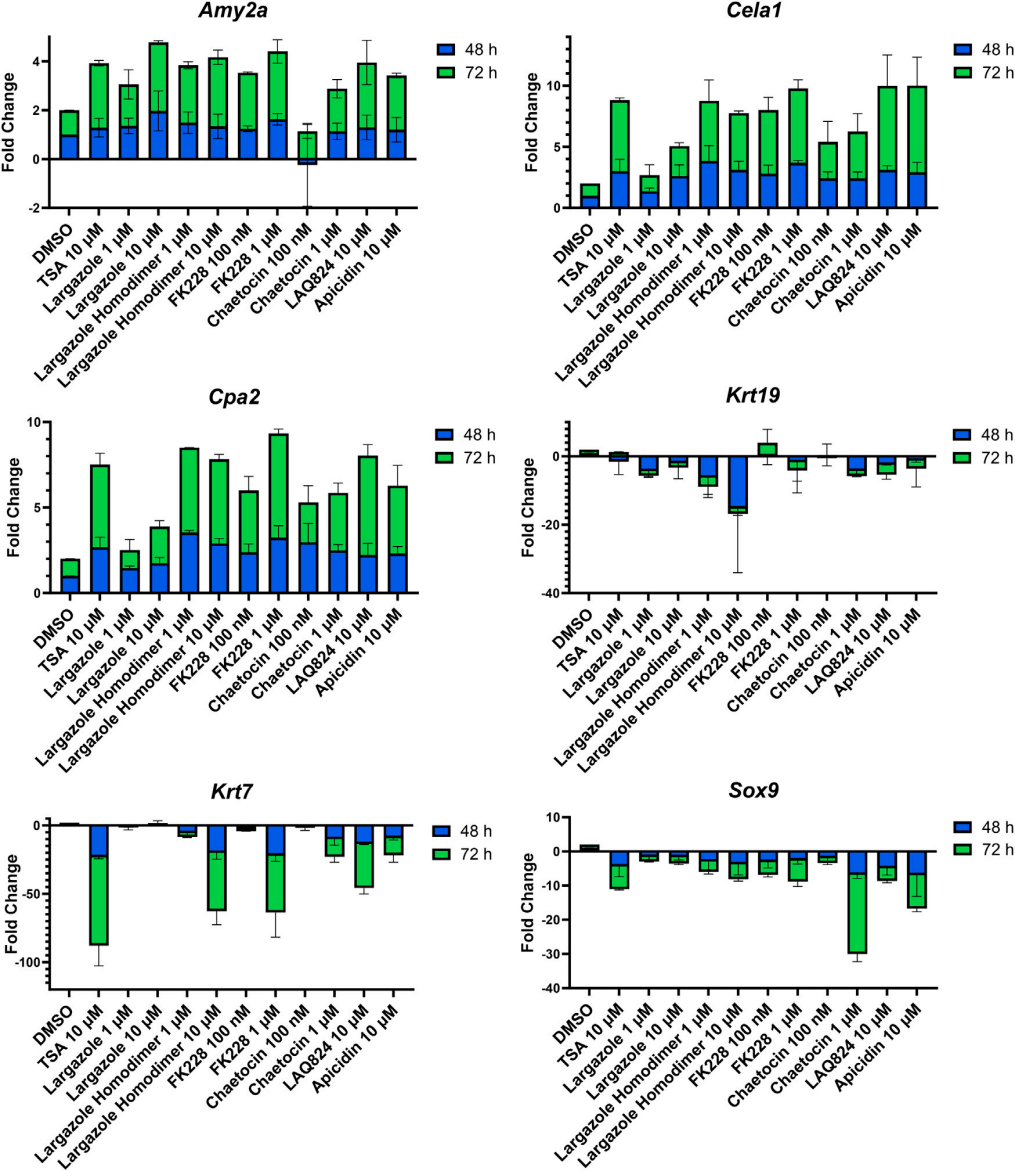
Validation of acinar and ductal gene expression following ADM reversal in KC organoids. KC mouse ADM organoids were treated with TSA, largazole, largazole homodimer, FK228, or chaetocin at varying concentrations for 2 or 3 days immediately following 2 days of ADM transdifferentiation. Cultures were collected following the 48 or 72 h treatments and gene expression was analyzed by qRT-PCR. Data are normalized to the 48 h DMSO control and are presented relative to 18S rRNA. Mean ± SD from two independent experiments.

qRT-PCR was used to validate that the morphological changes that occurred during ADM reversal of KC mouse organoids correlated to changes in acinar and ductal gene expression. Compared to DMSO control, all compounds tested produced increased expression of the acinar genes *Amy2a*, *Cela1*, and *Cpa2* with most occurring in a concentration-dependent fashion (Figure 12). Expression of the ductal genes were reduced by most of the treatments with largazole homodimer, FK228 and chaetocin demonstrating concentration-dependent reduction in *Krt19*, *Krt7*, and *Sox9* expression (Figure 12). The ADM reversal index (ADMRI) was used as a measure of drug effect with the greater magnitude of ADMRI indicating more ADM reversal. The data show that 1 μM FK228 was the most potent (ADMRI = 15.4) compound at reversing ADM while chaetocin and largazole dimer were comparatively less active at this concentration. LAQ824 and largazole dimer showed improved activity at 10 μM (ADMRI 15.9 and 15.1, respectively) with similar AMDRI values to FK228 at 1 μM, and largazole dimer being considerably more potent than its monomer (Table 2).

**TABLE 2:** ADMRI of lead compounds from KC reversal screening. An acinar to ductal metaplasia reversal index (ADMRI) was generated by dividing the mean fold change of the acinar genes by the mean fold change of the ductal genes to quantify the amount of molecular reversal that occurred in the cultures. A higher ADMRI value indicates a greater capability of reversing ductal cells back into their acinar state.

Compound	ADMRI (1 µM)	Ranking (1 µM)	ADMRI (10 µM)	Ranking (10 µM)
FK228	15.4	1	–	–
Chaetocin	11.7	2	–	–
Largazole homodimer	11.6	3	15.1	2
Largazole	2.0	4	2.6	5
LAQ824	–	–	15.9	1
Apicidin	–	–	14.6	3
TSA	–	–	9.1	4

– not tested.

**FIGURE 13:**
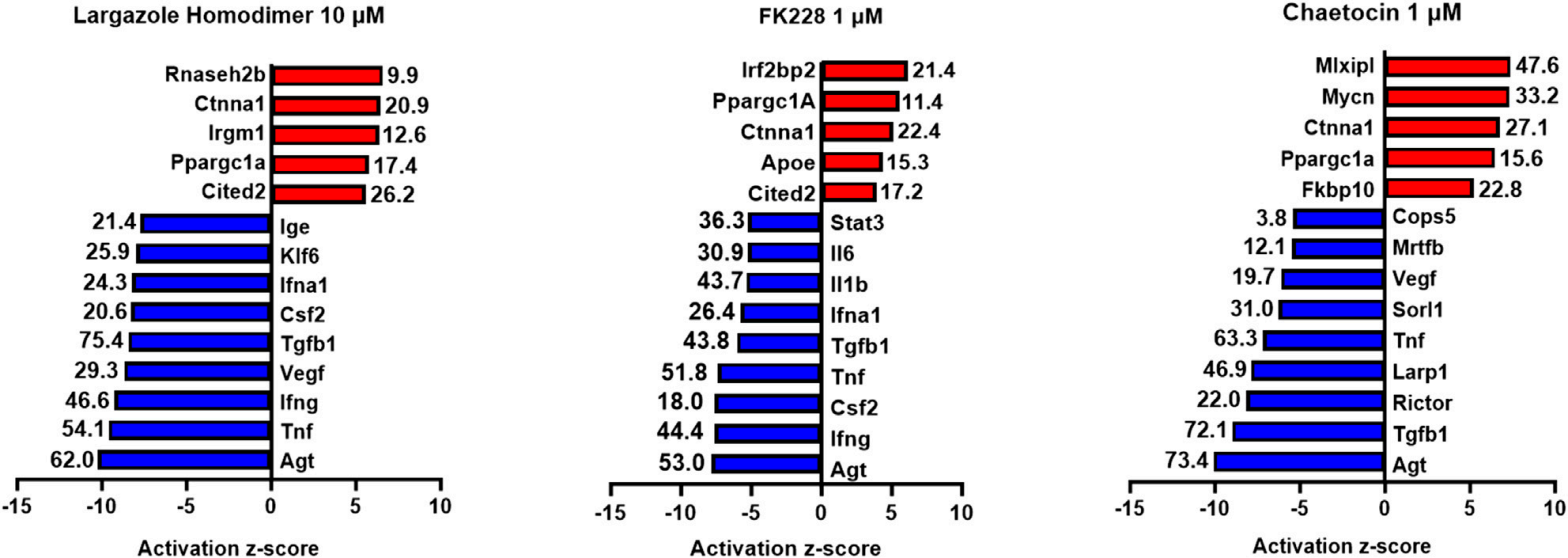
Ingenuity Pathway Analysis activation z-scores of regulated pathways from KC ADM reversal organoids. Ingenuity Pathway Analysis z-scores of top upregulated pathways from KC ADM reversal organoids. The RNA sequencing data from KC mice acinar organoids treated for ADM reversal with the compounds indicated were analyzed by Ingenuity Pathway Analysis (IPA) for the class of upstream regulators. The top upstream regulators are ranked by z-score with those regulators that are activated in red and those inhibited in blue. The -log (*p*-value) are indicated for each gene.

RNA obtained from the ADM reversal experiments in KC mice for the most potent compounds at an IC_90_ dose were submitted for RNA sequencing to further validate if differences in gene expression accompanied the morphological changes and to see if pathway analysis supports ADM reversal. The volcano plots of the drug-induced changes in gene expression show excellent correlations between acinar genes being upregulated and downregulation of ductal/PDAC genes upon reversal for largazole homodimer, FK228 and chaetocin (Supplementary Figure S6). Pathway analysis showed that the top inhibited upstream regulator for all three compounds was angiotensinogen (AGT). Other PDAC-related pathways that were inhibited for all three compounds during ADM reversal include TGFB1 and TNF. The pathways consistently activated for all three compounds during ADM reversal include α-catenin and PPARGC1A (Figure 13).

## 5 Discussion

We report the very first small molecule screen of its type for epigenetic regulators in pancreas. Our novel phenotypic small-molecule screen based on organoid morphology was used to discover epigenetic compounds that inhibit or reverse pancreatic ADM. Unlike anticancer agent screens that use cytotoxicity as an endpoint (George et al., 2010; Kim et al., 2018), quantification of acinar and ductal morphology using high-content imaging successfully identified compounds that inhibit or reverse ADM without inducing excessive cytotoxicity. For proof-of-concept, we utilized a focused library containing 144 epigenetic modulators with the goal of discovering effective compounds that act at the chromatin level. Using the HDAC inhibitor TSA as a positive control, reported in our prior work to inhibit and reverse ADM (da Silva et al., 2022), our robust screen produced acceptable Z′ scores ranging from 0.56 to 0.61.

Our strategy was to first screen the libraries on wildtype (inhibition) and Cre (reversal) mouse organoids then evaluate the top candidates for ADM reversal using the KC mouse cultures. The inhibition screens in wildtype mouse organoids produced two compounds that passed our rigorous selection criteria. These compounds include the HMT inhibitor chaetocin and the Zn^2+^-dependent, class I HDAC inhibitor apicidin (Figures 4, 5). Likewise, apicidin and chaetocin were the only two compounds selected to reverse ADM in the Cre mouse organoid screen (Figures 6, 7). To probe the involvement of class I HDAC isoforms, two additional class I HDAC inhibitors that were not part of the ESL (FK228 and largazole), were also evaluated for ADM reversal and inhibition in organoids that contain wildtype *Kras*, thereby linking the activity to HDACs 1-3. To summarize our findings in organoids with wildtype *Kras*, FK228 was the most effective compound at inhibiting and reversing ADM (low nM IC_50_) whereas apicidin, LAQ824 and chaetocin required somewhat higher concentrations to exert a similar effect (Figures 9, 10). We show that the stable homodimer of the natural product largazole (Taori et al., 2008; Liu et al., 2010) has ADM reversal effects and that alteration of the prodrug type can be used to modulate the activity profile (Figure 10).

In the ADM inhibition and reversal screens in organoids containing wildtype *Kras*, certain compounds induced unique morphological changes that were identified following ADM inhibition or reversal. Of note, four different BET inhibitors (IBET762, IBET151, (+) JQ1 and PFI-1) formed enlarged ducts during ADM inhibition. (Figure 8; Table 1). Bromodomain and extra-terminal (BET) proteins form complexes by binding to epigenetic marks such as acetylated lysine residues, reducing interaction between histones and DNA, thus increasing transcription. Nuclear expression of bromodomain containing 4 (BRD4), a BET protein important for enhancer-mediated transcription of cell-identity genes, was detected in normal acinar and duct cells and in the nucleus of acinar cells during ADM (Huang et al., 2016). Combining tissue injury with shRNA knockdown of BRD4 in wildtype mice or mice harboring a *Kras* mutation showed that BRD4-suppressed cells effectively lost their acinar morphology and acquired ductal markers; the authors concluded that BRD4 impairs ADM during normal regeneration (Alonso-Curbelo et al., 2021). Thus, it is reasonable to presume that the enlarged ducts formed by BRD4 inhibition of ADM in our experiments resulted from their inability to dedifferentiate.

A total of 7 compounds (Table 2) were selected for their ability to reverse ADM in the clinically relevant KC mouse organoid model. While ADM in the context of mutant *Kras* is believed to be irreversible, we previously showed using both morphology and gene expression analysis that TSA reverted KC mouse pancreatic ducts to an acinar state (da Silva et al., 2022). We used the same endpoints here to establish ADM reversibility in KC mouse organoids by epigenetic modulating compounds. The morphology data presented in Figure 11 showed somewhat mixed results. While higher concentrations of chaetocin reversed nearly all of the ducts to acinar clusters, the other compounds (apicidin, LAQ824, FK228 and largazole homodimer) induced modest levels of ADM reversal by morphology even at concentrations as high as 10 uM (Figure 11). Examination of the gene expression data revealed that nearly all of the compounds increased acinar and reduced ductal gene expression to some extent (Figure 12). We describe a new means to quantify the effectiveness of a compound to reverse ADM, the ADMRI (Equation 3.1). Presentation of the data by this index allowed us to rank the effectiveness of the top ranked compounds as determined by the selection criteria (Table 2). These data confirm that compounds selected for their ability to reverse ADM in wildtype mouse organoids reverse ADM in organoids containing mutant *Kras*. Moreover, our screening approach successfully identified compounds that are more active at reversing ADM compared to the TSA positive control (Table 2, 10 µM treatments) and invoked the class I HDACs as major functionally relevant targets. Precaution should be used when applying the ADMRI which is explicitly driven by both the upregulation of acinar genes and the downregulation of ductal genes, since the magnitude of the ADMRI might be directly impacted by large fold-change differences in only one of these values (e.g., no change in acinar expression but a large decrease in ductal expression).

It is noteworthy that in the KC mouse model, the top inhibited upstream regulator detected during ADM reversal (angiotensinogen, AGT) is the main precursor of angiotensin and part of the renin-angiotensin-aldosterone system (Figure 13). These data corroborate our findings of angiotensinogen as the most upregulated pathway during both human and mouse ADM (Jiang et al., 2023). Major components of the renin-angiotensin-aldosterone system (angiotensinogen and angiotensin receptors 1 and 2) were upregulated in rat pancreatic acinar cells during pancreatitis and treatment with the angiotensin receptor antagonist losartan inhibited the acinar digestion enzyme secretion (Huang et al., 2016). Moreover, PDAC patients who were prescribed angiotensin converting enzyme inhibitors or angiotensin receptor blockers to treat their hypertension were associated with better clinical outcomes compared to gemcitabine monotherapy (Nakai et al., 2010).

For organoids treated with the library at a final concentration of 1 μM, we identified and validated five compounds (apicidin, LAQ824, FK228, largazole/largazole dimer, and chaetocin) that induced complete or partial inhibition or reversal of ADM. Notably, of the five identified compounds, four (apicidin, LAQ824, FK228, and largazole) are Zn^2+^-dependent HDAC inhibitors, confirming the previously suggested importance of HDAC isoforms as possible targets for ADM modulation (da Silva et al., 2022; Liu et al., 2010; Taori et al., 2008). Our cumulative data indicate the importance of class I HDACs. FK228, and largazole specifically predominantly target HDACs 1, 2, and 3 and to a lesser extent HDAC8 (Hong and Luesch, 2012). The tested library contained a total of 34 Zn^2+^-dependent HDAC (class I/II/IV) inhibitors with broad or class-specific selectivity and 9 NAD^+^-dependent (class III) HDAC inhibitors, raising questions as to why the other class I HDAC inhibitors did not show similar effects as apicidin, LAQ824, FK228, and largazole. This could be due to the limitations of the current screen which used 1 μM as the primary screening concentration, selected based on a cytotoxicity screen of the ESL using cancer cell lines (data not shown). The 1 μM screening concentration ensured that over 80% of the tested compounds displayed less than 50% cytotoxicity, but it may fall below the IC_50_ of some of the compounds. Other possible reasons for the small amount of hits detected in this focused library screen could be a narrow target specificity, structure-specific physicochemical issues, or other differential downstream mechanisms involved that should be further explored. We have previously observed compound-specific effects of HDAC inhibitors in another small-molecule screen for epigenetic modulators of nuclear morphology (Atanasova et al., 2022).

We used the CellProfiler Analyst software for object classification of acinar clusters *versus* ducts for cell counting. Over the years, CellProfiler has presented challenges for researchers in cell segmentation due to its insufficient ability to detect numerous cell types, especially if interconnected (Stirling et al., 2021). When our screening efforts began, CellProfiler was the most optimal, automated means for object classification. With the emergence of artificial intelligence, AI-mediated approaches for cluster *versus* duct classification may be used to address the caveats of CellProfiler.

## 6 Conclusion

In conclusion, we developed a novel phenotypic drug screen using organoid morphology as a readout to discover epigenetic regulator compounds with unique mechanism of action (i.e., ADM inhibition and reversal). Validation of the top hits (FK228, chaetocin, LAQ824, and largazole homodimer) in organoids derived from a clinically relevant KC mouse model confirmed that ADM can be reversed without inducing significant cytotoxicity even in the presence of mutant *Kras*. Our findings demonstrate a unique mechanism of action for epigenetic compounds and suggest that the phenotypic screen developed here may be applied to discover potential new treatments for PDAC.

## Data Availability

The data presented in the study are deposited in the https://www.ncbi.nlm.nih.gov/ repository, accession number GSE236292.
